# Endophytic Bacteria and Fungi Associated with *Polygonum cuspidatum* in the Russian Far East

**DOI:** 10.3390/plants13182618

**Published:** 2024-09-19

**Authors:** Olga A. Aleynova, Alexey A. Ananev, Nikolay N. Nityagovsky, Andrey R. Suprun, Nursaule Zh. Zhanbyrshina, Alina A. Beresh, Zlata V. Ogneva, Alexey P. Tyunin, Konstantin V. Kiselev

**Affiliations:** 1 Laboratory of Biotechnology, Federal Scientific Center of the East Asia Terrestrial Biodiversity, Far Eastern Branch of the Russian Academy of Sciences, 690022 Vladivostok, Russia; ananev.all@yandex.ru (A.A.A.); niknit1996@gmail.com (N.N.N.); a.beresh@mail.ru (A.A.B.); zlata.v.ogneva@gmail.com (Z.V.O.); tyunin@biosoil.ru (A.P.T.); kiselev@biosoil.ru (K.V.K.); 2 The Department of Agriculture and Plant Growing, S. Seifullin Kazakh Agrotechnical Research University, Astana 010011, Kazakhstan; 3 Institute of the World Ocean, Far Eastern Federal University, 690090 Vladivostok, Russia

**Keywords:** Asian knotweed, endophyte diversity, *Fallopia japonica*, Japanese knotweed, microbiome, next-generation sequencing, *Reynoutria japonica*

## Abstract

*Polygonum cuspidatum*, alternatively known as *Fallopia japonica* or *Reynoutria japonica*, is a perennial herb belonging to the Polygonaceae family. Commonly called Japanese knotweed or Asian knotweed, this plant is native to East Asia, particularly in regions such as Korea, China, and Japan. It has successfully adapted to a wide range of habitats, resulting in it being listed as a pest and invasive species in several countries in North America and Europe. This study focuses on analysing the composition of the bacterial and fungal endophytic communities associated with Japanese knotweed growing in the Russian Far East, employing next-generation sequencing (NGS) and a cultivation-based method (microbiological sowing). The NGS analysis showed that the dominant classes of endophytic bacteria were Alphaproteobacteria (28%) and Gammaproteobacteria (28%), Actinobacteria (20%), Bacteroidia (15%), and Bacilli (4%), and fungal classes were Agaricomycetes (40%), Dothideomycetes (24%), Leotiomycetes (10%), Tremellomycetes (9%), Pezizomycetes (5%), Sordariomycetes (3%), and Exobasidiomycetes (3%). The most common genera of endophytic bacteria were *Burkholderia*-*Caballeronia*-*Parabukholderia*, *Sphingomonas*, *Hydrotalea*, *Methylobacterium*-*Metylorubrum*, *Cutibacterium*, and *Comamonadaceae*, and genera of fungal endophytes were *Marasmius*, *Tuber*, *Microcyclosporella*, *Schizothyrium*, *Alternaria*, *Parastagonospora*, *Vishniacozyma*, and *Cladosporium*. The present data showed that the roots, leaves, and stems of *P. cuspidatum* have a greater number and diversity of endophytic bacteria and fungi compared to the flowers and seeds. Thus, the biodiversity of endophytic bacteria and fungi of *P. cuspidatum* was described and analysed for the first time in this study.

## 1. Introduction

*Polygonum cuspidatum* Sieb. and Zucc. [1], also known as *Fallopia japonica* or *Reynoutria japonica* (Polygonaceae), is a robust, herbaceous perennial that produces annual, smooth, tubular stems that rise from an upright base. These stems typically exhibit a light green colour, often accompanied by reddish spots, and can grow to heights of up to 3 m [1]. *P. cuspidatum* is classified as an invasive species on numerous lists, including the IUCN’s list of the top 100 worst invasive species. Due to its ability to thrive in different soil types and environments, it poses a significant threat as a soil contaminant and has the potential to spread widely. Known for its resilience in breaking through tough structures and its difficulty to eradicate once established, it is considered one of the most persistent weeds in any new habitat [2].

*P. cuspidatum* is rich in flavonoids, anthraquinones, and stilbenes. Resveratrol, polydatin, quercetin, emodin, and their analogues are the main bioactive compounds found in *P. cuspidatum* [3,4]. Among numerous plants that contain resveratrol, *P. cuspidatum* is the most prevalent [5]. These natural compounds have been extensively studied and are believed to play a crucial role in the medicinal properties of *P. cuspidatum* [6,7]. *P. cuspidatum*-based derivatives display a broad spectrum of medicinal properties, including anti-inflammatory, antioxidant, antitumour, cardioprotective, and other therapeutic effects. In medical practice, it is prescribed for conditions such as vertigo, migraines, physical trauma, and thermal injuries [2]. At present, the roots of this species are used in Russia as a raw material for the production of biologically active additives due to their high content of resveratrol and polydatin. These biologically active additives are used in antibacterial, antiviral, antiparasitic, antifungal, immunostimulating, immunomodulating, and anti-inflammatory therapies.

Endophytes, which include a wide array of microorganisms such as fungi, bacteria, algae, and actinomycetes, inhabit plant tissues without inflicting harm to their host [8]. They play an essential role in the host plant’s ecosystem and can affect metabolic pathways, promoting the synthesis of bioactive compounds that hold medicinal and agricultural importance [9]. Endophytic organisms have the potential to provide new and valuable natural products for the pharmaceutical and agricultural industries [10]. A large number of endophytes have the ability to synthesise secondary plant compounds that are similar to those found in their host plants [10,11].

For example, the *P. cuspidatum* endophytic actinomycete *Streptomyces* sp. A0916 exhibits a broad spectrum of antimicrobial properties, demonstrating superior effectiveness compared to extracts from its host *P. cuspidatum* extracts [12]. Also, Jiewei and colleagues [13] showed that resveratrol, when fermented with *Streptomyces* sp. A12, is transformed to 3,5,4′-trimethoxy-*trans*-stilbene, which demonstrated the ability to induce cell cycle arrest [14] and apoptosis [15] through different mechanisms of action, unlike resveratrol, and occasionally, 3,5,4′-trimethoxy-*trans*-stilbene showed better potency and efficacy than resveratrol [16]. The endophytic fungus *Penicillium* sp., obtained from *P. cuspidatum*, is capable of converting resveratrol into *trans*-3,5-dimethoxy-4′-hydroxystilbene, commonly referred to as pterostilbene. Pterostilbene, a dimethylated form of resveratrol, has greater biological activity, increased membrane permeability, and improved metabolic stability compared to its original form. This makes pterostilbene a promising candidate for the treatment of various human diseases, positioning it as the next-generation resveratrol with significant pharmacological potential [17]. Further, the conversion of resveratrol to pterostilbene for enhanced stability and bioefficiency was accomplished by endophytic *Penicillium* sp. sourced from *P. cuspidatum* [17,18]. Various studies have been conducted to optimise the bioconversion of polydatin to resveratrol, with *Aspergillus niger* and yeast successfully transforming polydatin into resveratrol in *P. cuspidatum* roots [12]. Additionally, *Dekkera bruxellensis* exhibited promising glycosidic-linked resveratrol hydrolysis activity in *P. cuspidatum*, leading to a significant increase in resveratrol production [19]. Endophytic bacteria, such as *Bacillus aryabhattai*, isolated from the rhizome tissue of *Reynoutria japonica* (*P. cuspidatum*), along with *Bacillus safensis*, have also demonstrated an ability to convert polydatin into resveratrol [20,21]. The presence of endophytic fungi in *P. cuspidatum* has a positive impact on plant development and increases the concentration of bioactive compounds [22]. Six new guanacastane diterpenes Cercosporenes A-F (1–6, respectively) were obtained from the unrefined extract of the fungus *Cercospora* sp., residing within herb *Fallopia japonica*. Along with showing cytotoxic effects on various human tumour cell lines, such as HeLa, A549, MCF-7, HCT116, and T24, heterodimer 6 also induced autophagy in HCT116 cells [23].

Therefore, studying the biodiversity and properties of endophytes of invasive plants, including *P. cuspidatum*, is an interesting and promising task. To date, the biodiversity of Japanese knotweed endophytes has been poorly described. Kurose et al. isolated fungal endophytes from *F. japonica* in its natural habitat in Japan and recovered 15 taxa. The analysis revealed that five genera, *Alternaria*, *Colletotrichum*, *Pestalotiopsis*, *Phoma*, and *Phomopsis*, were the predominant endophytes associated with *F. japonica* [24]. Previously, *Alternaria* and *Colletotrichum* were also dominant genera isolated from *P. cuspidatum* in China, while *Pestalotiopsis* and *Phoma* were not recorded [25]. Our research investigated the bacterial and fungal endophytes associated with *P. cuspidatum* in the Russian Far East using next-generation sequencing (NGS) and a cultivation-based method (microbiological sowing).

## 2. Results

### 2.1. Illumina MiSeq Sequencing

Employing next-generation sequencing (NGS), 5,270,947 *16S* and 2,007,201 *ITS1* paired-end reads were acquired from 58 *Polygonum cuspidatum* plant specimens (6–8 specimens from each plant). Following paired-end alignments, quality filtration, and removal of chimeric, mitochondrial, chloroplast, *Viridiplantae*, *Metazoa*, *Rhizaria*, *Protista*, *Alveolata*, and unidentifiable sequences, a total of 1,991,232 *16S* and 372,535 *ITS1* sequences were produced (Tables S1 and S2). In terms of the *16S* data, the average and median sequence counts for the samples were found to be 34,332 and 37,444, respectively. Meanwhile, for the *ITS1* data, the average and median read counts were 6423 and 3880, respectively.

### 2.2. General Composition of Endophytic Community in Different Polygonum cuspidatum Organs

**Figure 1:**
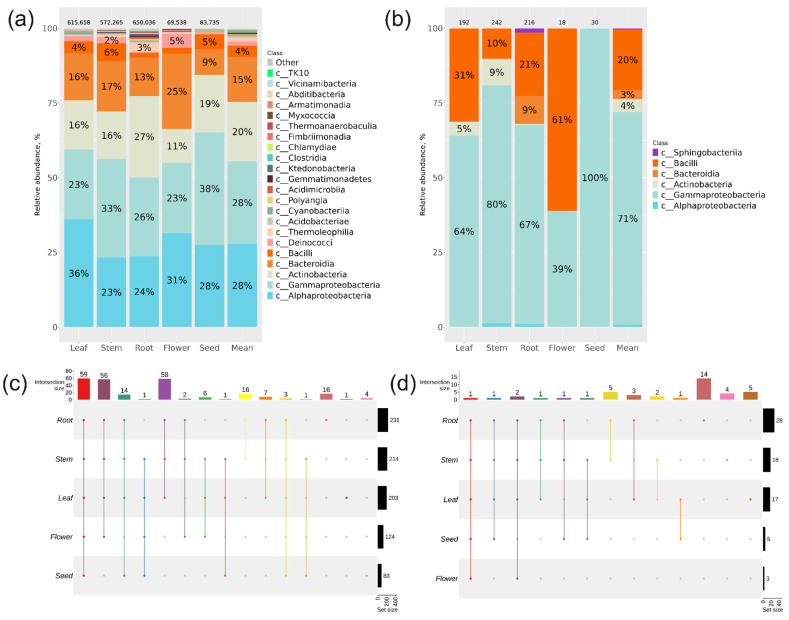
Comparison of the bacterial endophytic communities in various organs of *Polygonum cuspidatum* was conducted using two different approaches: next-generation sequencing (NGS) and a cultivation-based method (microbiological sowing). Endophytic bacteria composition in *P. cuspidatum* varied depending on the specific organ of the plant: (**a**) Class-level taxonomical bar plots were generated for the community of bacteria obtained through NGS in roots, stems, leaves, flowers, seeds, and the average data for all organs; (**b**) Class-level taxonomical bar plots were created for the bacterial community from bacteriological sowing in roots, stems, leaves, flowers, seeds, and the mean data for all organs. (**c**,**d**) Genus-level UpSet diagrams illustrated the overlapping taxa from NGS and bacteriological sowing in different organs, respectively. Taxa with a relative abundance of >0.1% were included in the analysis, with any filtered taxa placed in the “other” category on the bar plot and omitted from the UpSet diagram. The number of colonies (for sowing) or sequences above the taxonomical bar plots was indicated.

Based on the metagenomic analysis of *16S* sequences, a total of 245 taxa at the genus level with a relative abundance above 0.1% were identified in the community of endophytic bacteria of different *P. cuspidatum* organs (Figure 1c, Table S3). These genera were grouped into 22 taxa at the class level (Figure 1a).

The prevailing bacterial classes were Alphaproteobacteria (28%) and Gammaproteobacteria (28%), Actinobacteria (20%), Bacteroidia (15%), and Bacilli (4%) (Figure 1a). Interestingly, the highest percentage of Gammaproteobacteria was found in *P. cuspidatum* seeds (38%) and stem (33%) (Figure 1a). In addition to the five main dominant classes, the Thermoleophilia (3%) and Deinococci (5%) classes were also relatively well represented in *P. cuspidatum* roots and flowers, respectively (Figure 1a).

Furthermore, a cultivation-based method (bacteriological sowing) was utilised to investigate the endophytic bacteriome of *P. cuspidatum* utilising sterilised plant tissues. A total of 698 bacterial isolates were analysed following the microbiological inoculation procedure carried out for different organ samples, and these isolates were classified into four major bacterial classes: Gammaproteobacteria was the dominant class at 71%, followed by Bacilli at 20%, Actinobacteria at 4%, and Bacteroidia at 3% (Figure 1b).

The analysis of the endophytic bacteriome of the different organs of *P. cuspidatum* showed that the highest number of genus-level taxa was identified in roots, followed by leaves and stems, while seeds and flowers displayed a much smaller variety of bacterial endophytes (Figure 1c,d). In particular, a total of 59 were detected in all *P. cuspidatum* organs by NGS (Figure 1c, Table S3), and only one common genus, *Pseudomonas*, was typical of all organs by the cultivation-dependent approach (Figure 1d, Table S4). Sixteen and fourteen root-specific endophytic bacterial genera were found using the two approaches (Figure 1c,d, Tables S3 and S4). One and four unique bacterial genera were detected in leaf and seed samples by NGS (Figure 1c, Table S3).

**Figure 2:**
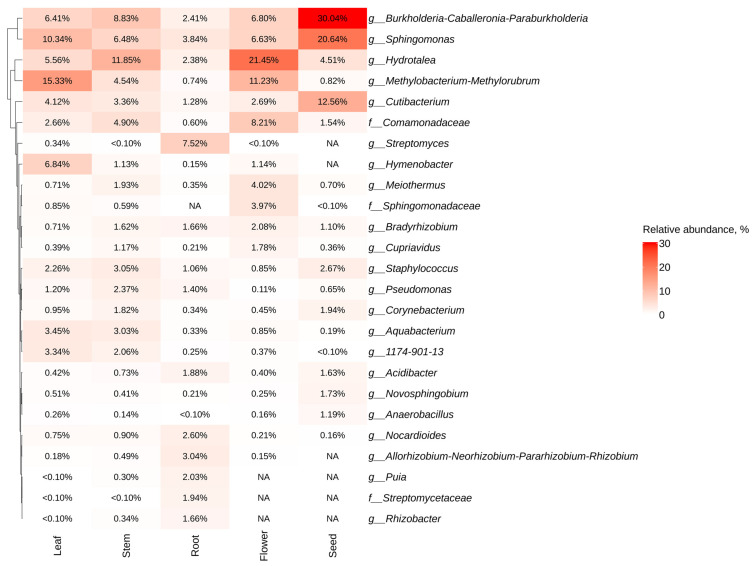
Heat maps illustrating the relative abundance of endophytic bacteria at the genus level were constructed, focusing on the significant taxa identified through next-generation sequencing (NGS) across various organs of *Polygonum cuspidatum* (root, stem, leaves, flower, and seed). The top 10 taxa with the highest abundance in each factor are shown. Absence of taxa is indicated by white squares (NA).

The most common genera for all *P. cuspidatum* organs were the taxa *Burkholderia-Caballeronia-Parabukholderia*, *Sphingomonas*, *Hydrotalea*, *Methylobacterium-Metylorubrum*, *Cutibacterium*, and *Comamonadaceae* sp. (Figure 2). The most prevalent genera found in the *P. cuspidatum* roots were *Streptomyces* (7.5%), *Sphingomonas* (3.8%), *Allorhizobium-Neorhizobium-Pararhizobium-Rhizobium* (3%), *Nocardiodes* (2.6%), and *Puia* (2%), while in the flowers, the dominant species were *Hydrotalea* (21.5%), *Methylobacterium*-*Methylorubrum* (11.2%), and *Comamonadaceae* (8.2%) (Figure 2). The highest number of sequences in *P. cuspidatum* seeds were attributed to the taxa *Burkholderia-Caballeronia-Parabukholderia* (30%). In addition, the taxa *Puia*, *Streptomycetaceae*, and *Rhizobacter* were represented in all organs except flowers and seeds (Figure 2).

**Figure 3:**
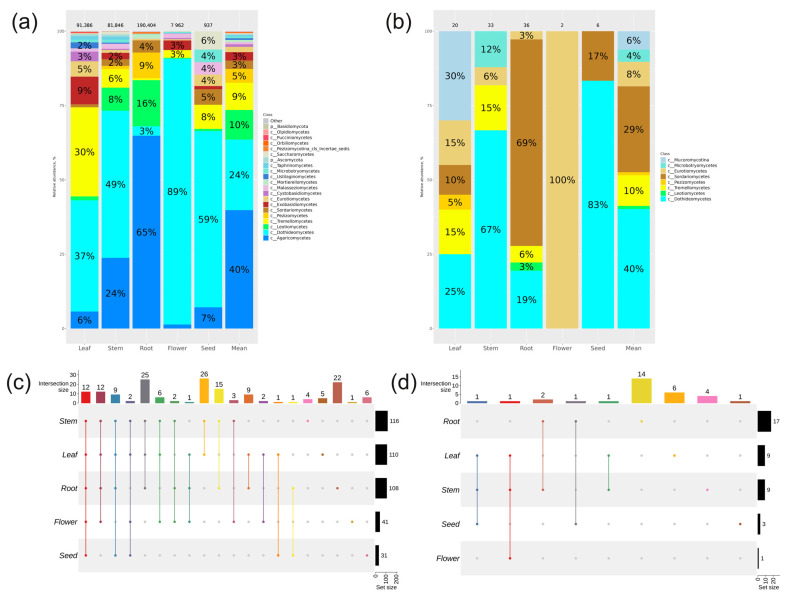
Comparison of the fungal endophytic communities in various organs of *Polygonum cuspidatum* was conducted using two different approaches: next generation sequencing (NGS) and a cultivation-based method (microbiological sowing). Endophytic fungal composition in *P. cuspidatum* varied depending on the specific organ of the plant: (**a**) Class-level taxonomical bar plots were generated for the fungal community obtained through NGS in roots, stems, leaves, flowers, seeds, and the mean data for all organs; (**b**) Class-level taxonomical bar plots were created for the fungal community from microbiological sowing in roots, stems, leaves, flowers, seeds, and the mean data for all organs. (**c**,**d**) Genus-level UpSet diagrams illustrated the overlapping taxa from NGS and microbiological sowing in different organs, respectively. Taxa with a relative abundance of >0.1% were included in the analysis, with any filtered taxa placed in the “other” category on the bar plot and omitted from the UpSet diagram. The number of colonies (for sowing) or sequences above the taxonomical bar plots was indicated.

According to the NGS analysis of *ITS1* sequences, 21 taxa of class level were found in various *P. cuspidatum* organs with a relative abundance above 0.1%. Of the 21 taxa, sequences of two fungal classes were the most prevalent: Agaricomycetes at 40% and Dothideomycetes at 24% (Figure 3a). The classes Exobasidiomycetes, Sordariomycetes, Pezizomycetes, Tremellomycetes, and Leotiomycetes were represented in the mycobiome of Japanese knotweed from 3 to 10% (Figure 3a). In the roots of *P. cuspidatum*, the dominant class of endophytic fungi was the class Agaricomycetes (65%), followed by Leotiomycetes (16%) and Pezizomycetes (9%) (Figure 3a). The largest proportion of the Tremellomycetes class (30%) was found in the leaves of *P. cuspidatum*, whereas 1–8% of this class was found in the rest of the Japanese knotweed.

A total of 164 taxa at the genus level were found in various organs of *P. cuspidatum* (Figure 3c, Table S5). The largest number of genera was found in stems, followed by leaves and roots. Flowers and seeds had the smallest number of genus-level taxa (Figure 3c, Table S5). Unique genera of endophytic fungi, characteristic of only one organ, were found in roots (22), leaves (5), stems (4), seeds (6), and flowers (1) (Figure 3c, Table S5).

Ninety-seven strains of *P. cuspidatum* endophytic fungi were isolated by microbiological seeding. All these strains were representatives of eight fungal classes: the dominant class was Dothideomycetes at 40%, followed by Sordariomycetes, Tremellomycetes, Eurotiomycetes, Mucoromycotina, Microbotryomycetes, Leotiomycetes, and Pezizomycetes (Figure 3b). The largest number of strains was obtained from roots and stems, while only six and two endophytic fungi were isolated from seeds and flowers, respectively (Figure 3b). Fourteen root-specific endophytic fungi were found. Six, four, and one unique genera were found in leaf, stem, and seed, respectively (Figure 3d, Table S6).

**Figure 4:**
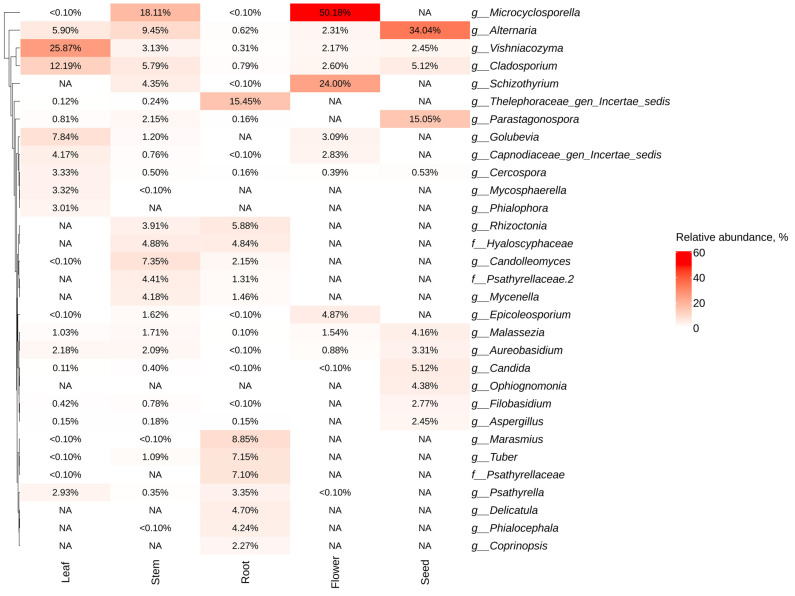
Heat maps illustrating the relative abundance of endophytic fungi at the genus level were constructed, focusing on the significant taxa identified through next-generation sequencing (NGS) across various organs of *Polygonum cuspidatum* (root, stem, leaves, flower, and seed). The top 10 taxa with the highest abundance in each factor are shown. Absence of taxa is indicated by white squares (NA).

It is worth noting that each organ of *P. cuspidatum* had its own unique composition of endophytic fungal genera (Figure 4). For example, the fungal genus-level taxa *Thelephoraceae*, *Marasmius*, *Tuber*, and *Psathyrellaceae* were most abundant in the root, while these taxa were absent from the flowers and seeds (Figure 4). The most common genera for the flowers were *Microcyclosporella* and *Schizothyrium*, while the most dominant genera for the seeds were represented by *Alternaria* and *Parastagonospora* (Figure 4). The dominant genera of endophytic fungi were *Vishniacozyma* and *Cladosporium* for leaves and *Microcyclosporella* and *Alternaria* for stems (Figure 4).

**Figure 5:**
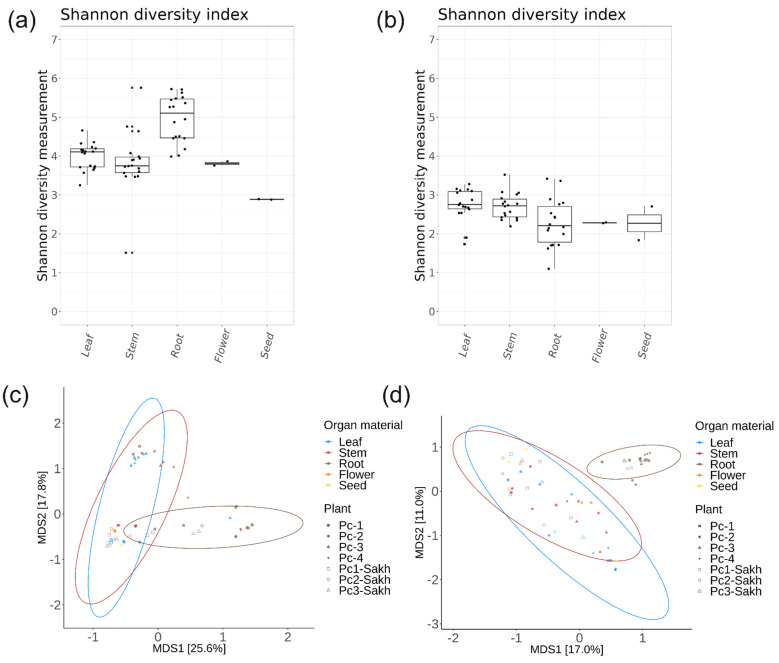
The comparative analyses of endophytic bacterial (**a**,**c**) and fungal (**b**,**d**) communities in different *Polygonum cuspidatum* organs. (**a**,**b**) Shannon alpha diversity boxplots; (**c**,**d**) Bray–Curtis beta diversity PCoA plots.

The amplicon data from each organ sample were analysed in relation to the plant part. The bacterial *16S P. cuspidatum* samples from the roots were statistically different (*p* < 0.05) in terms of alpha diversity compared to the other organs, while the alpha diversity of the *ITS1* samples was not statistically different (Figure 5b, Table S7). Compared to the other organ samples, the Shannon diversity index of endophytic bacteria was higher for root samples (Figure 5a). According to the PCoA ordination plots of beta diversity, bacterial and fungal communities in leaf, stem, flower, and seed samples form clusters that overlap to a high degree with each other, while root samples form a more separate cluster (Figure 5c,d). The PERMANOVA analysis indicated that the variation attributed to different organs accounted for 21% of the discrepancies observed in the bacterial endophytic community of *P. cuspidatum* samples (*p* = 0.001). In contrast, this organ factor contributed to 17% of the variation noted in the fungal endophytic community (*p* = 0.001) (Table S8).

### 2.3. Seasonal Variation in Endophytic Community Composition of Polygonum cuspidatum

**Figure 6:**
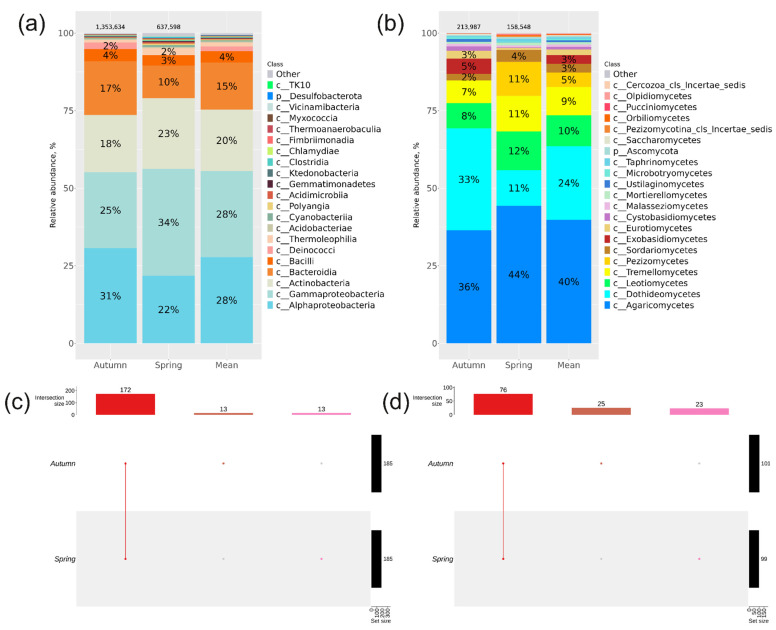
Composition of the endophytic community in *Polygonum cuspidatum* according to season of sampling: (**a**,**b**) taxonomic bar plots at class level for the bacterial and fungal community analysed by next-generation sequencing (NGS) in spring and autumn; (**c**,**d**) UpSet plots at the bacterial and fungal genus levels showing the overlap of taxa from NGS in spring and autumn. For every biocompartment, taxa were selected based on a relative abundance of over 0.1%. Taxa exhibiting a relative abundance below 0.1% were excluded from the UpSet plot. The count of sequences is displayed above the taxonomic bar graphs.

Based on the collection season, the biodiversity profile of endophytic bacteria and fungi in *P. cuspidatum* was investigated. Roots, leaves, and stems of *P. cuspidatum* were gathered in the second half of May, and additional roots, leaves, stems, flowers, and seeds were collected in the second half of September. A total of 636,598 *16S* sequences and 158,548 *ITS1* sequences were obtained in the spring, and 1,353,634 *16S* sequences and 213,987 *ITS1* sequences were obtained in the autumn of 2023 using NGS (Figure 6). The distribution between the classes of endophytic bacteria was different in spring and autumn (Figure 6a). In autumn, the percentages of the Alphaproteobacteria and Bacteroidia classes increased due to a decrease in the percentages of the Gammaproteobacteria and Actinobacteria classes. The composition of the endophytic fungi varied depending on the season. In the spring samples of *P. cuspidatum*, in addition to the dominant class Agaricomycetes, the classes Dothideomycetes, Leotiomycetes, Tremellomycetes, and Pezizomycetes were represented in almost equal proportions (11–12%) (Figure 6b), whereas in the autumn samples, the percentages shifted towards the class Dothideomycetes (36%), and the classes Exobasidiomycetes (5%) and Eurotiomycetes (3%) were also more represented than in the spring samples (Figure 6b).

In spring and autumn, 198 and 124 common genera of bacteria and fungi were found in *P. cuspidatum*, respectively (Figure 6c,d, Tables S9 and S10). Thirteen bacterial genera were unique for the spring season, and thirteen were unique for the autumn season. Unique *P. cuspidatum* genera were more abundant in autumn (25 genera) than spring (23 genera) (Figure 6c,d, Tables S9 and S10).

**Figure 7:**
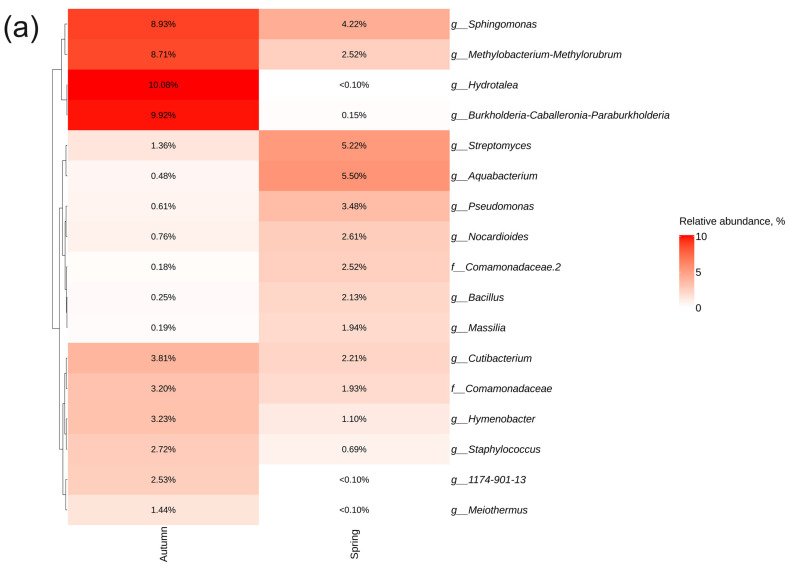

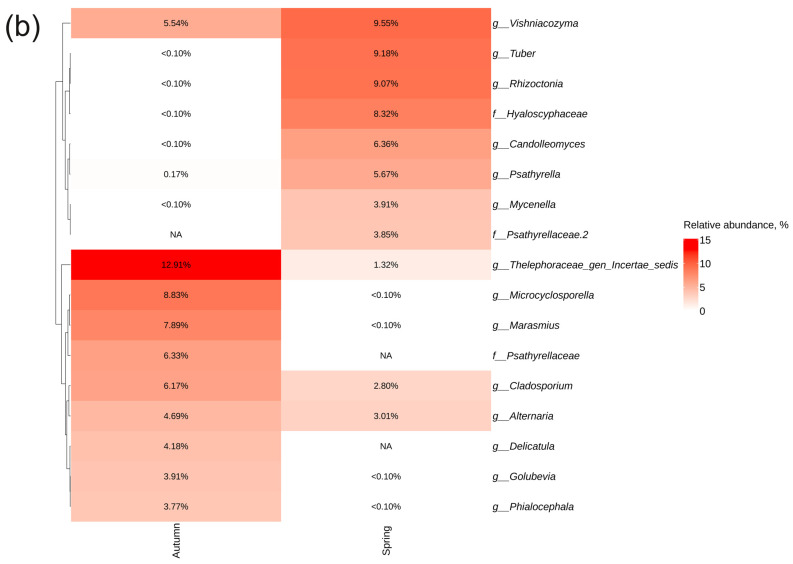
Heat maps illustrating the relative abundance of endophytic (**a**) bacteria and (**b**) fungi at the genus level were constructed, focusing on the significant taxa identified through next-generation sequencing (NGS) across different sampling seasons of *Polygonum cuspidatum*. The top 10 taxa with the highest abundance in each factor are shown. Absence of taxa is indicated by white squares (NA).

The most abundant bacterial genera in spring were *Aquabacterium* (5.5%), *Streptomyces* (5.2%), and *Sphingomonas* (4.2%), and the most abundant fungal genera were *Vishniacozyma* (9.6%), *Tuber* (9.2%), and *Rhizoctonia* (9.2%) (Figure 7), whereas in autumn, the bacterial genera *Hydrotalea* (10%), *Burkholderia*-*Caballeronia*-*Paraburkholderia* (9.9%), and *Methylobacterium*-*Methylorubrum* (8.7%) and the fungal taxa *Thelephoraceae* (13%), *Microcyclosporella* (8.8%), and *Marasmius* (7.9%) were dominant (Figure 7).

**Figure 8:**
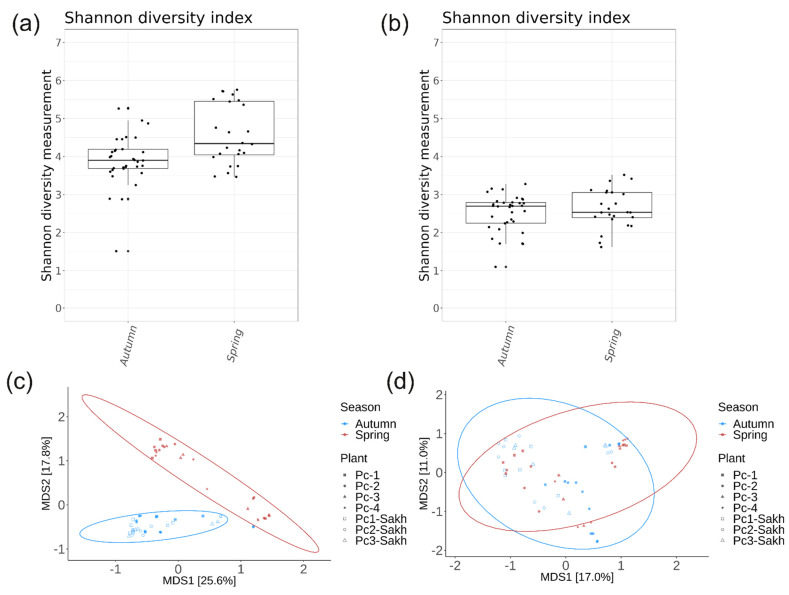
Comparative analyses of endophytic bacterial (**a**,**c**) and fungal (**b**,**d**) communities of *Polygonum cuspidatum* depending on the season of material collection. (**a**,**b**) Boxplots of Shannon alpha diversity; (**c**,**d**) PCoA plots of Bray–Curtis beta diversity.

Alpha and beta diversity analyses were also performed on the *16S* and *ITS1* amplicon data, depending on the season of collection (Figure 8a,b). The Shannon diversity index for bacterial endophytes was higher in spring samples compared to autumn samples (*p* = 0.0045) (Figure 8a). *P. cuspidatum* samples collected in both seasons were not statistically different in terms of fungal alpha diversity (*p* = 0.61) (Figure 8b). PCoA ordination showed that the spring and autumn samples in the bacterial community were located in separate clusters (Figure 8c), while the clusters of both seasons in the fungal community had a high overlap (Figure 8d). The PERMANOVA analysis revealed that seasonal variations were responsible for 10% of the differences within the bacterial endophytic community (*p* = 0.001), while this seasonal factor only accounted for 5% of the variances in the fungal endophytic community (*p* = 0.001), as detailed in Table S8.

### 2.4. Endophyte Community Composition in Different Polygonum cuspidatum Representatives

Furthermore, we compared the percentages of the bacterial and fungal communities within individual plants of *P. cuspidatum*. Four representatives of *P. cuspidatum* plants were growing in Primorsky Territory (Pc-1, Pc-2, Pc-3, Pc-4), and two samples were located on Sakhalin Island (Pc1-Sakh, Pc2-Sakh, Pc3-Sakh).

**Figure 9:**
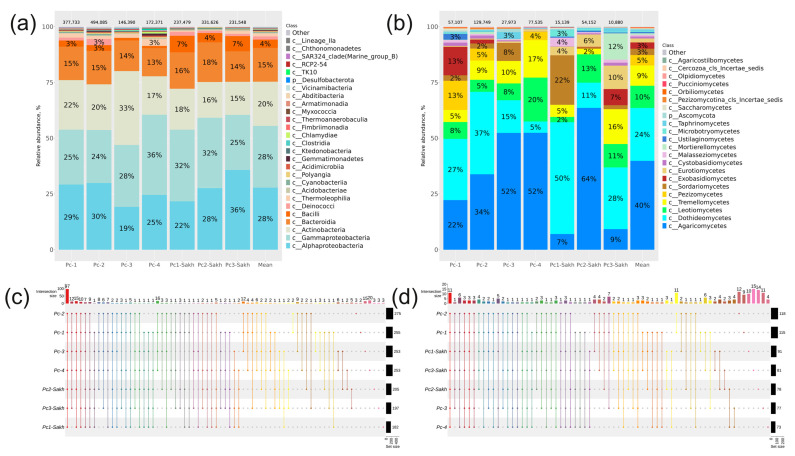
Composition of the endophytic community in six different *Polygonum cuspidatum* plants: (**a**,**b**) taxonomic bar plots at class level for the community of bacteria and fungi analysed by next-generation sequencing (NGS); (**c**,**d**) UpSet plots at the bacterial and fungal genus levels showing the overlap of taxa from NGS. For every biocompartment, taxa were selected based on a relative abundance of over 0.1%. Taxa exhibiting a relative abundance below 0.1% were excluded from the UpSet plot. The count of sequences is displayed above the taxonomic bar graphs.

The percentage of endophytic bacteria did not vary significantly between individual plants and sampling sites (Figure 9a). The biodiversity of endophytic bacteria in *P. cuspidatum* samples collected in Primorsky Territory was represented by 253–275 bacterial genera, while samples collected on Sakhalin Island were represented by 182–205 bacterial genera. Ninety-seven genera of endophytic bacteria were common to all *P. cuspidatum* plants (Figure 9c, Table S11).

However, depending on the collection site and the individual plant, the percentage of fungal endophytes varied considerably. Thus, in the Pc1-Sakh sample, the proportion of the Dothideomycetes class reached 50% (the average value in all samples for this class is 24%), and the proportion of the Agaricomycetes class was 7% (against an average of 40%). The percentages of the Sordariomycetes class (22%) and the Malasseziomycetes class (4%) were also relatively increased in the Pc1-Sakh sample (Figure 9b). Only 11 genera of endophytic fungi were common to all plants collected (Figure 9d, Table S12).

**Figure 10:**
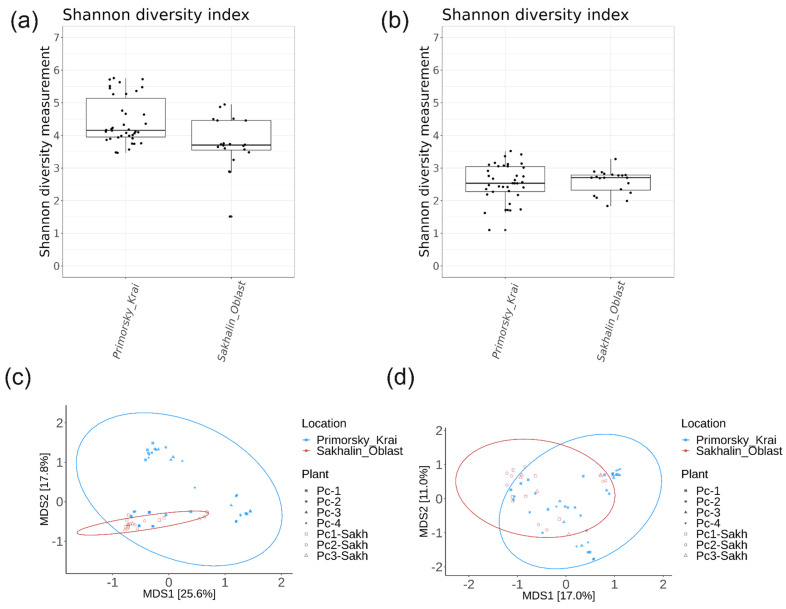
Comparative analyses of endophytic bacterial (**a**,**c**) and fungal (**b**,**d**) communities of *Polygonum cuspidatum* depending on the localisation of material collection. (**a**,**b**) Boxplots of Shannon alpha diversity; (**c**,**d**) PCoA plots of Bray–Curtis beta diversity.

We conducted an analysis of the amplicon data from each sampling site concerning the occurrence of *P. cuspidatum*. Sakhalin Island samples have a lower alpha diversity of bacterial endophytes compared to those from Primorsky Territory (*p* = 0.0013) (Figure 10a). Samples collected in Primorsky Territory and Sakhalin Island were similar in terms of alpha diversity of fungal endophytes (*p* = 0.78) (Figure 10b). The PCoA ordination plot showed that samples from Primorsky Territory and Sakhalin Island were in clusters with a high overlap (Figure 10c,d). The PERMANOVA test indicated that the location variable accounted for 6% of the variance in the bacterial endophytic community among *P. cuspidatum* samples (*p* = 0.001). Similarly, this variable also contributed to 6% of the variance in the fungal endophytic community across samples (*p* = 0.001) (Table S8).

## 3. Discussion

*Polygonum cuspidatum* is an important agricultural plant due to its high concentration of valuable medicinal compounds (such as polydatin, resveratrol, chrysophanol, and emodin) [22]. A number of studies have shown that endophytic microorganisms can have a significant effect on the levels of valuable secondary metabolites in Japanese knotweed [22,26]. Moreover, endophytes can play a crucial role in adapting plants to challenging environments, such as drought, heat, and heavy metal contamination. Therefore, studying the biodiversity of the endophytic community in *P. cuspidatum* may be an important task to increase the content of important secondary metabolites in this plant.

This research aimed to analyse the microbial communities present in the roots, stems, leaves, seeds, and berries of *P. cuspidatum* to reveal the concealed majority of these internal microbial populations and to achieve a deeper comprehension of the endophytic microbial communities associated with this plant. Bacterial populations appear to be more abundant than fungal populations within Japanese knotweed. This is consistent with the data obtained from the *16S* rRNA sequencing reads and subsequent analysis.

During the analysis of the endophytic community of *P. cuspidatum* bacteria using two approaches (culture-dependent and culture-independent), the main classes of bacteria were Alphaproteobacteria and Gammaproteobacteria, Actinobacteria, Bacteroidia, and Bacilli, which indicates the reliability of the results obtained. When analysing the endophytic community of *P. cuspidatum* fungi, the main classes were Dothideomycetes, Sordariomycetes, Tremellomycetes, Eurotiomycetes, Mucoromycotina, Microbotryomycetes, Leotiomycetes, and Pezizomycetes, which was confirmed by the two approaches used. However, the dominant fungi using the NGS method were Agaricales, which were not detected at all using cultivation methods. It is known that some Agaricales species are difficult to grow and that media other than PDA may be more selective. Therefore, the use of methods to analyse the endophytic community (dependent and independent of cultivation) gives a more complete picture of the endophytic community of plants.

The Bray–Curtis beta diversity PCoA plot shows the differences in the sequencing of bacteria and fungi between the root and aboveground plant organs of *P. cuspidatum* (Figure 5). Factors such as plant age and tissue type influence the diversity of endophytic microorganisms in plants [27,28]. Also, such a strong difference in the endophytic composition is due to the physiological structure and nutritional components of the individual organs of *P. cuspidatum*, namely the composition and content of sugars, secondary metabolites, Ph, etc. Root endophytic bacteria are more diverse than those found in stems and leaves (Figure 5), probably because the majority of these bacteria originate from soil in the rhizosphere [12]. It was found that the most common root genera were *Streptomyces* and *Nocardiodes*. Earlier, it was shown that *Streptomyces* sp. A0916 extracts from *P. cuspidatum* exhibited broad-spectrum antimicrobial activity and greater antimicrobial efficacy than the *P. cuspidatum* extracts [12]. There are also papers mentioning that some strains of *Streptomyces* bacteria are able to convert valuable metabolites, for example, converting resveratrol into 3, 5, 4′-trimethoxy-trans-stilbene [13]. Perhaps finding a relatively large number of *Streptomyces* sp. in the roots of Japanese knotweed makes biological sense, for example, by protecting *P. cuspidatum* plants from an excess of resveratrol by modifying it into a less active substance or an evolutionary way of protecting *Streptomyces* with other competing microorganisms. It has also been shown that some strains of *Nocardioides* bacteria are able to biodegrade volatile chlorinated compounds (particularly vinyl chloride), which are the main pollutant of ground water and create a risk of vapor penetration into buildings [29]. The presence of a relatively high percentage of this genus may indicate the presence of volatile chlorinated compounds in the soil where the *P. cuspidatum* samples were collected.

Among the endophytic fungi, the most common taxa found in roots were represented by the families *Thelephoraceae* and *Psathyrellaceae* and the genera *Marasmius* and *Tuber*. Recent studies have shown that some species of *Marasmius oreades* have nematocidal and insecticidal activity through the production of agglutinins, which could be exploited for crop protection from these important agricultural pests [30]. Perhaps the presence of this endophyte in the roots of *P. cuspidatum* is also an evolutionary symbiosis, resulting in a mutually beneficial relationship that arises between the endophytic fungus and the plant host. Further research on specific species of the genus *Marasmius* may contribute to the development of new biopesticides that can be used in the agricultural sector.

The most abundant taxa of endophytic bacteria in the terrestrial organs of *P. cuspidatum* were *Burkholderia*-*Caballeronia*-*Parabukholderia* and *Sphingomonas*. Some bacteria of the genus *Burkholderia* are able to synthesise antimicrobial secondary metabolites that are active against fungi [31,32]. In addition, enzymes from some *Sphingomonas* species have a high efficiency in converting polydatin to resveratrol [33]. Also, various types of the endophytic bacteria *Methylobacterium* have the ability to convert nitrogen, form nodules in the host plant, and synthesise cytokinins, auxins, and enzymes associated with stimulating overall plant resistance, such as pectinase and cellulase, thereby enhancing plant development. These bacteria can also be used to minimise environmental pollution through their ability to degrade harmful substances, withstand elevated levels of heavy metals, and increase plant resistance to such substances [34].

One of the most common endophytic genera of *P. cuspidatum* fungi in all organs was the genus *Alternaria*. This genus is one of the significant plant pathogens. Also, it is well known that some strains of *Alternaria* are capable of resveratrol synthesis [35] and that their extracts have cytotoxic effects [36]. Also, some representatives of *Parastagonospora*, which had a high occurrence in *P. cuspidatum* seeds, in particular *Parastagonospora nodorum*, stand out as important necrotrophic pathogens of wheat, causing considerable economic damage to crop production [37]. In addition, *P. cuspidatum* flowers contained relatively high levels of *Microcyclosporella* and *Schizothyrium* fungi. Some species of the genus *Microcyclosporella* are known to cause sooty blotch and flyspeck defects on the surface of pomaceous fruits, particularly apples [38]. *Schizothyrium* fungi can cause sooty blotch and flyspeck on apple fruit [39,40]. Therefore, some of the endophytic microorganisms of Japanese knotweed may be potential pathogens of crop plants.

On the other hand, the leaves of *P. cuspidatum* had a high proportion of *Vishniacozyma* yeasts. Some species of *Vishniacozyma* are known to be able to increase host resistance to the fruit pathogen *Botrytis cinerea* [41]. Thus, Japanese knotweed contains endophytic bacteria and fungi that can be both pathogens and of practical importance for the protection of agricultural crops and for the biochemical transformation of stilbenes.

Also, the biodiversity of Japanese knotweed endophytes is directly dependent on environmental conditions, namely humidity and environmental temperature, as well as the growth stage of the plant. In spring, the plant only produces stems and leaves, and the young tissues are probably colonised by more frost-resistant species, which in autumn give way to more competitive but more capable endophytes. We found that the spring samples had the highest diversity of the endophytic bacterial community, according to the Shannon diversity index. The diversity of *P. cuspidatum* endophytes is also likely to vary significantly between different geographical locations, in particular Primorsky Territory and Sakhalin Island. This variation can be attributed to the unique evolutionary history of the *P. cuspidatum* endophyte community, which has been shaped over decades by the specific soil conditions and climatic factors of each region. Geographical location also influences endophytic communities, as different regions have unique microclimates, soil types, and plant communities, all of which can shape the composition of endophytes.

In conclusion, the endophytic bacterial and fungal communities of *P. cuspidatum* of the Russian Far East have been analysed for the first time using an integrated approach, namely NGS and microbiological seeding. The findings from this study contribute to bridging the existing gap in scientific knowledge regarding the endophytic microorganisms of *P. cuspidatum*. Nonetheless, further comprehensive studies focusing on plant–endophyte interactions are essential for the future. We highlight the capacity of *P. cuspidatum* endophytes or their bioactive compounds as potential solutions to combat emerging diseases in agricultural crops and the problem of antibiotic resistance. Furthermore, *P. cuspidatum* endophytic microorganisms can be used as a new tool for highly efficient and clean production of resveratrol.

## 4. Materials and Methods

### 4.1. Collection of Polygonum cuspidatum Samples

Samples of three *Polygonum cuspidatum* plants (average leaves, steams, roots, flowers, and seeds) were collected from a nonprotected natural population near Vladivostok, Russia (Pc-1—43.167747, 131.918894; Pc-2—43.165629, 131.919144; Pc-3—43.175195, 131.912495), and samples of one *P. cuspidatum* plant were harvested at the Verkhne-Ussuriysky Research Station (SSA) of the Federal Scientific Center of the East Asia terrestrial biodiversity Far East Branch of the Russian Academy of Sciences (Pc-4—43.695667, 132.156544) in May and September 2023; additionally, samples of two *P. cuspidatum* plants were collected on Sakhalin Island, Russia (Pc-Sakh-1—46.854018, 142.571455 and Pc-Sakh-2—47.057867, 142.130470) in September 2023 using sterile instruments. The roots were extracted with the soil and placed in a sterile bag; then, the roots were cleaned from the soil under laboratory conditions. Each plant sample was transferred to the laboratory in sterile bags within one day at 10 °C and used for DNA and endophyte isolation. The data from these three sites were used for the analysis of the composition of the bacterial and fungal endophytes.

The average temperature and precipitation in Vladivostok (Pc-1, Pc-2, Pc-3) and the central part of Primorsky Territory at the SSA (Pc-4) were 16–18 °C and 120–125 mm in the second part of May 2023 and 19 °C and 50 mm in September 2023 (https://world-weather.ru/pogoda/russia/vladivostok/june-2023/, accessed on 28 May 2024). On Sakhalin Island (Pc-Sakh-1 and Pc-Sakh-2), the mean temperature and precipitation in September 2023 were 21 °C and 150 mm (https://world-weather.ru/pogoda/russia/yuzhno_sakhalinsk/september-2023/, accessed on 28 May 2024). Within 1–2 days, each plant sample was delivered to the laboratory in sterile bags.

Plant material for genomic approaches and classical microbiological techniques (bacterial and fungal seeding) was collected in May and September. Roots, stems, leaves (May and September), and seeds and berries (September) were sampled from each plant. In total, 58 biological replicates of *P. cuspidatum* were collected and analysed. In addition, 2 technical replicates were used for each biological replicate.

### 4.2. Isolating and Identifying Polygonum cuspidatum Bacterial and Fungal Endophytes

Each sample of *P. cuspidatum* (1.5 g) underwent surface sterilisation according to previously described methods [42,43]. To verify the effectiveness of the sterilisation process, 100 µL of the last rinsing water was cultured on potato dextrose agar (PDA, Neogene, Watford, UK) and R2A (PanReac, AppliChem, Darmstadt, Germany) plates to confirm the absence of external colony growth. The sterilised *P. cuspidatum* tissues were homogenised in a sterile mortar, and the resulting juice was plated on R2A and PDA plates. Bacterial and fungal colonies were isolated and subcultured for further analysis after 2 and 7 days. In total, 698 bacterial and 89 fungal endophytic strains were identified.

The hexadecyltrimethylammonium bromide (CTAB) method with modifications was used to extract DNA from different strains of endophytes [44]. Bacterial *16S* rRNA gene sequences and fungal *ITS1* PCR products were amplified using universal bacterial and fungal primers [45,46]. Sequencing of the PCR products was performed and analysed as previously described [47]. A sequence similarity of ≥99% was considered adequate for taxonomic identification at the genus level [48].

### 4.3. DNA Extraction from Polygonum cuspidatum and Preparation of Library for Use on an Illumina MiSeq Sequencing System

DNA was isolated for NGS using the CTAB spin method, as previously described, from leaves, stems, roots, flowers, and seeds of *P. cuspidatum* plants [42,43,49]. The DNA samples were sent to Syntol company in Moscow, Russia for advanced sequencing using Illumina technology. The quality and quantity of the DNA were assessed using the Nanodrop-1000 (Nanodrop, Wilmington, NC, USA) and Quantus Fluorometer (Promega, Madison, WI, USA), respectively. The sequencing libraries were prepared according to a detailed protocol provided by the manufacturer of the sequencing technology “*16S* Metagenomic Sequencing Library Preparation” (Part # 15,044,223 Rev. B; Illumina). Bacterial *16S* rRNA regions and fungal *ITS1* rDNA regions were amplified from all samples using the modified plant primers described earlier [42,43]. The Nextera^®^ XT Index Kit (Illumina, San Diego, CA, USA) was used to index the amplicons. The library pool underwent sequencing on the Illumina MiSeq (Illumina, San Diego, CA, USA) platform (2 × 250 paired end), utilising MiSeq reagent kit v2 (Illumina, San Diego, CA, USA) with 500 cycle paired-end reads.

The sequences of the bacterial and fungal endophytes have been deposited at the NCBI under the accession number PRJNA1127768 and in the database of laboratory Biotechnology, Federal Scientific Center of the East Asia Terrestrial Biodiversity, Far Eastern Branch of the Russian Academy of Sciences, Russia (https://biosoil.ru/downloads/biotech/Metagenoms/2023-10-seq=Illumina=6(Reynoutria)/) (accessed on 19 July 2024).

### 4.4. Data Processing

The data for bioinformatic analysis are available in Tables S1 and S2. The custom scripts in R and Bash were used to process the data (https://github.com/niknit96/Aleynova_et.al.2024/, accessed on 10 September 2024). Paired-end reads were preprocessed using QIIME 2 [50] and DADA2 [51] to remove primers, PhiX reads, and chimeric sequences. The reads were then merged and sorted. The taxonomic identification of sequences was performed using the QIIME 2 Scikit-learn method with the SILVA 138 pre-trained classifier for *16S* sequences (99% OTUs from V4 region) [52] and UNITE pre-trained classifier for *ITS* sequences (99% OTUs from ITS1f/ITS2 region) [53].

The qiime2R [54], phyloseq [55], RColorBrewer [56], circlize [57], and tidyverse [58] R libraries were used to filter and prepare the data. Amplicon sequence variants were merged into taxonomic ranks at the genus level. Mitochondria, chloroplast, non-bacterial, and non-fungal sequences were deleted from the obtained data. Taxa for bar plots and UpSet diagrams were filtered based on a relative abundance of >0.1% for each factor. In the bar plots, we merged the taxonomic ranks that had a relative abundance <0.1% in each factor to one group called “other”. In the heat maps, the top 10 taxa with the highest abundance are shown for each factor. Shannon alpha diversity and Bray–Curtis beta diversity data were obtained using the phyloseq [55] and microViz [59] R packages, respectively. Bray–Curtis dissimilarity data were presented as principal coordinates analysis (PCoA) ordination plots. A pairwise Wilcoxon rank-sum test with the false discovery rate correction method was performed to analyse the alpha diversity data between groups. Statistical validation of beta diversity data was performed using the PERMANOVA test with 999 permutations. The ggplot2 [58], microViz [59], and ComplexHeatmap [60] R libraries were used to graphically represent the results.

## Data Availability

The data presented in this study are available within the article and Supplementary Material.

## Supplementary Materials

The following are available online at https://www.mdpi.com/article/10.3390/plants13182618/s1, Table S1. *16S* data samples of *Polygonum cuspidatum* used in analysis (BioProject: PRJNA1127768); Table S2. *ITS* data samples of *Polygonum cuspidatum* used in analysis (BioProject: PRJNA1127768); Table S3. Overlapping genus-level taxa in the *16S* metagenomic data of different organs of *Polygonum cuspidatum*; Table S4. Overlapping genus-level taxa in the *16S* cultivation-dependent data of different organs of *Polygonum cuspidatum*; Table S5. Overlapping genus-level taxa in the *ITS* metagenomic data of different organs of *Polygonum cuspidatum*; Table S6. Overlapping genus-level taxa in the *ITS* cultivation-dependent data of different organs of *Polygonum cuspidatum*; Table S7. Pairwise comparisons using the Wilcoxon rank-sum exact test on Shannon diversity data from different organs of *Polygonum cuspidatum*; Table S8. Results of statistical validation of beta diversity data using the PERMANOVA test with 999 permutations; Table S9. Overlapping genus-level taxa in *16S* metagenomic data from *Polygonum cuspidatum* across seasons; Table S10. Overlapping genus-level taxa in *ITS* metagenomic data from *Polygonum cuspidatum* across seasons; Table S11. Overlapping genus-level taxa in *16S* metagenomic data from *Polygonum cuspidatum* across plants; Table S12. Overlapping genus-level taxa in *16S* metagenomic data from *Polygonum cuspidatum* across plants.
